# An Oxidative Stress Index-Based Score for Prognostic Prediction in Colorectal Cancer Patients Undergoing Surgery

**DOI:** 10.1155/2021/6693707

**Published:** 2021-01-09

**Authors:** Yinghao Cao, Shenghe Deng, Lizhao Yan, Junnan Gu, Fuwei Mao, Yifan Xue, Changmin Zheng, Ming Yang, Hongli Liu, Li Liu, Qian Liu, Kailin Cai

**Affiliations:** ^1^Department of Gastrointestinal Surgery, Union Hospital, Tongji Medical College, Huazhong University of Science and Technology, Wuhan, Hubei 430022, China; ^2^School of Optical and Electronic Information, Huazhong University of Science and Technology, Wuhan 430074, China; ^3^Department of Pathology, Union Hospital, Tongji Medical, Huazhong University of Science and Technology, Wuhan, Hubei 430022, China; ^4^Cancer Center, Union Hospital, Tongji Medical College, Huazhong University of Science and Technology, Wuhan 430022, China; ^5^Department of Epidemiology and Biostatistics, the Ministry of Education Key Lab of Environment and Health, School of Public Health, Tongji Medical College, Huazhong University of Science and Technology, Wuhan, Hubei 430022, China; ^6^Department of Cardiology, Wuhan Women and Children Medical Care Center, Wuhan, Hubei 430022, China

## Abstract

Oxidative stress plays an important role in the development of colorectal cancer (CRC). This study is aimed at developing and validating a novel scoring system, based on oxidative stress indexes, for prognostic prediction in CRC patients. A retrospective analysis of 1422 CRC patients who underwent surgical resection between January 2013 and December 2017 was performed. These patients were randomly assigned to the training set (*n* = 1022) or the validation set (*n* = 400). Cox regression model was used to analyze the laboratory parameters. The CRC-Integrated Oxidative Stress Score (CIOSS) was developed from albumin (ALB), direct bilirubin (DBIL), and blood urea nitrogen (BUN), which were significantly associated with survival in CRC patients. Furthermore, a survival nomogram was generated by combining the CIOSS with other beneficial clinical characteristics. The CIOSS generated was as follows: 0.074 × albumin (g/L), −0.094 × bilirubin (*μ*mol/L), and -0.099 × blood urea nitrogen (mmol/L), based on the multivariable Cox regression analysis. Using 50% (0.1025) and 85% (0.481) of CIOSS as cutoff values, three prognostically distinct groups were formed. Patients with high CIOSS experienced worse overall survival (OS) (hazard ratio [HR] = 4.33; 95% confidence interval [CI], 2.80-6.68; *P* < 0.001) and worse disease-free survival (DFS) (HR = 3.02; 95% CI, 1.96-4.64; *P* < 0.001) compared to those with low CIOSS. This predictive nomogram had good calibration and discrimination. ROC analyses showed that the CIOSS possessed excellent performance (AUC = 0.818) in predicting DFS. The AUC of the OS nomogram based on CIOSS, TNM stage, T stage, and chemotherapy was 0.812, while that of the DFS nomogram based on CIOSS, T stage, and TNM stage was 0.855. Decision curve analysis showed that these two prediction models were clinically useful. CIOSS is a CRC-specific prognostic index based on the combination of available oxidative stress indexes. High CIOSS is a powerful indicator of poor prognosis. The CIOSS also showed better predictive performance compared to TNM stage in CRC patients.

## 1. Introduction

Colorectal cancer (CRC) is the third most common cancer, accounting for 9.7% of all cancers except melanoma skin cancer. It is the second and third most common cancer in women and men, respectively, accounting for 10% of all new cancer cases each year [1]. The “rise” of CRC in developed countries can be attributed to an ageing population, unfavorable modern dietary habits, and an increase in risk factors such as smoking, inadequate physical activity, and obesity [2]. Despite significant advances in treatment strategies, the mortality rate from CRC remains high; from 1990 to 2013, the death rate from CRC increased by 57%. In 2013, there were 771,000 deaths from CRC, which now accounts for about 10% of the cancer-related deaths worldwide [3]. At present, the long-term prognosis of CRC patients is still not optimistic, warranting a reasonable and effective prediction model for the long-term prognostic assessment of CRC patients.

Integrating different types of information into an accurate, personalized CRC prognostic assessment tool is a challenge, but few assessments of the internal or external effectiveness of prognostic models are currently conducted [4]. At present, the common prognostic indicators of CRC patients include age, tumor location, tumor stage, tumor marker level, and presence of metastasis. Although CRC prognosis has been proved to be closely related to the aforementioned indicators, they have not been effectively verified externally and their prediction accuracy is also not high [5–7]. Considering that quite a few factors are closely related to the prognosis of CRC patients, a reasonable, simple, accurate, and low-cost prediction model should be designed to effectively predict the prognosis of patients.

In addition, oxidative stress, mainly associated with mutations in the colorectal genes, has an equally important predictive role in the occurrence, development, and prognosis of CRC [8]. Studies have also shown that some plant-based foods with the potential to inhibit inflammation and oxidative stress may be beneficial to CRC patients, especially those whose molecular markers point to severe inflammation and oxidative stress [9]. A multicenter randomized controlled study has found that a healthy diet has an important role in suppressing inflammation and oxidative stress in long-term disease outcome and survival in patients with CRC [10]. Given the critical role of oxidative stress in the progression of CRC, we attempted to investigate the prognostic significance of oxidative stress-related indexes in this study.

In the present study, we first created and validated a new CRC-Integrated Oxidative Stress Score (CIOSS) from a large sample of CRC patients undergoing surgical operation. Furthermore, we compared the predictive value of CIOSS and TNM staging system for the prediction of OS and DFS. Finally, we combined the new indicators with other common clinical variables to create and validate sequence diagrams for predicting OS and DFS in patients with CRC.

## 2. Materials and Methods

### 2.1. Patients and Study Design

This clinical study was performed at the Wuhan Union Hospital with a total of 1422 CRC patients who underwent surgical resection between January 2013 and December 2017. The inclusion criteria were as follows: (1) confirmed CRC diagnosis by abdominal computed tomography (CT) or biopsy, (2) underwent surgical resection with no evidence of distant metastasis, and (3) availability of complete clinical and pathological data. Patients with the following conditions were excluded from the study: (1) history of tumor, coinfection, or blood disease; (2) treatment with anti-inflammatory drugs prior to surgical resection; (3) presence of severe cardiovascular disease or metabolic diseases; (4) without clinical and follow-up information; (5) received chemotherapy or pharmacological treatment before surgery.

A total of 1422 surgically resected CRC patients with complete clinical and follow-up data were included in this study and randomly divided into the training (*n* = 1022) and validation (*n* = 400) groups. This study was approved by the ethics committee of Wuhan Union Medical College Hospital (No. 2018-S377) and was carried out in accordance with the Helsinki Declaration. All patients signed an informed consent regarding their understanding of the procedure and its potential complications, as well as their approval for participation in the research.

### 2.2. Data Collection

Baseline clinicopathologic data obtained from hospital medical records included age, gender, smoking status, tumor history, intestinal obstruction, tumor differentiation, tumor size, tumor location, tumor T stage, tumor N stage, tumor TNM stage, perineural invasion, vascular invasion, and chemotherapy. Tumor stage was classified according to the 8^th^ edition of the American Joint Committee on cancer staging system, and patients with high-risk stages II or above received adjuvant chemotherapy postoperation.

Follow-up data were obtained every three months, and whenever we suspected recurrence, gastroscopy and imaging were performed. The primary endpoint of this study was OS, and the secondary endpoint was DFS. OS was defined as the time interval from surgery to the last follow-up or death. DFS was defined from the date of definitive surgery to the date of first recurrence (local or distant) or the date of last follow-up.

### 2.3. Evaluation of the Oxidative Stress Indexes

Routine blood and biochemistry tests were conducted from the first day of admission for each CRC patient. The oxidative stress indexes in our study comprised ALB, total bilirubin (TBIL), DBIL, BUN, and uric acid (UA). The cutoff values of these oxidative stress indexes were identified using the X-tile software [11].

### 2.4. Statistical Analysis

Statistical analysis was performed using the SPSS 23.0 (SPSS Inc., Chicago, IL, USA) and R 4.0.0 software (Institute for Statistics and Mathematics, Vienna, Austria). Continuous data were expressed as mean ± standard deviation, unless otherwise specified. Patient characteristics were compared using *t*-tests for continuous variables and *χ*^2^ or Fisher exact tests for categorical variables. X-tile 3.6.1 (Yale University, New Haven, CT, USA) was used to determine the optimal cutoff value for ALB, TBIL, DBIL, BUN, and UA levels. The training set was applied to develop the novel oxidative stress score based on the *β* coefficients from the multivariable Cox analysis [12]. CRC patients were divided into three risk groups (low risk, intermediate risk, and high risk) based on the 50^th^ and 85^th^ percentiles of CIOSS. Receiver operating characteristic (ROC) analyses were then performed to assess the predictive abilities of CIOSS and TNM stage for the prediction of survival outcomes. Moreover, univariate and multivariate Cox proportional hazards regression models were utilized to evaluate the prognostic factors for OS and DFS. A nomogram incorporating the important factors related to the OS or DFS was constructed with the R software. Td-ROC curves were drawn to assess the predictive performance of survival nomogram for 1-year, 3-year, and 5-year prediction. To further measure the calibration of the nomogram in predicting survival, a calibration curve was generated to compare the observed results with the predicted results. A value of *P* < 0.05 was considered significant.

## 3. Results and Discussion

### 3.1. Study Population

**Figure 1:**
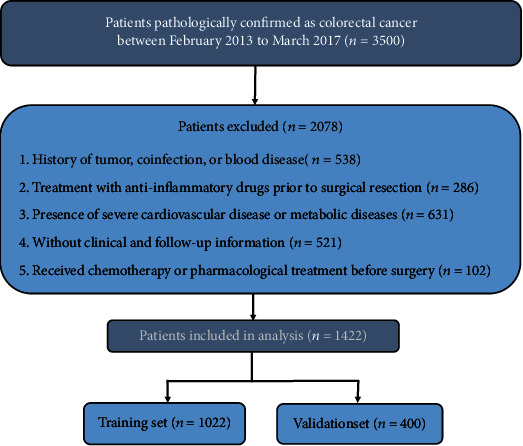
Strategies for selecting patients to be included in the study.

A total of 1422 participants from Wuhan Union Hospital were included in this research; 825 were males, 597 were females, and mean age was 57.79 ± 11.26 years (range, 20-85). Of these, 70% (*n* = 1022) of the patients were randomly assigned to the training set while the remaining patients (*n* = 400) were included in the internal validation set. A detailed flow diagram of the patient selection process is shown in Figure 1. The clinical characteristics of CRC patients in the training and validation set are given in Table 1. The cutoff values for oxidative stress were determined using the X-tile software. Based on these cutoff values, all oxidative stress indicators included in our study were those that statistically correlated with OS (Fig. S1 A-E) and DFS (Fig. S1 F-J) in CRC patients.

### 3.2. Creation of the Novel Oxidative Stress Index

**Figure 2:**
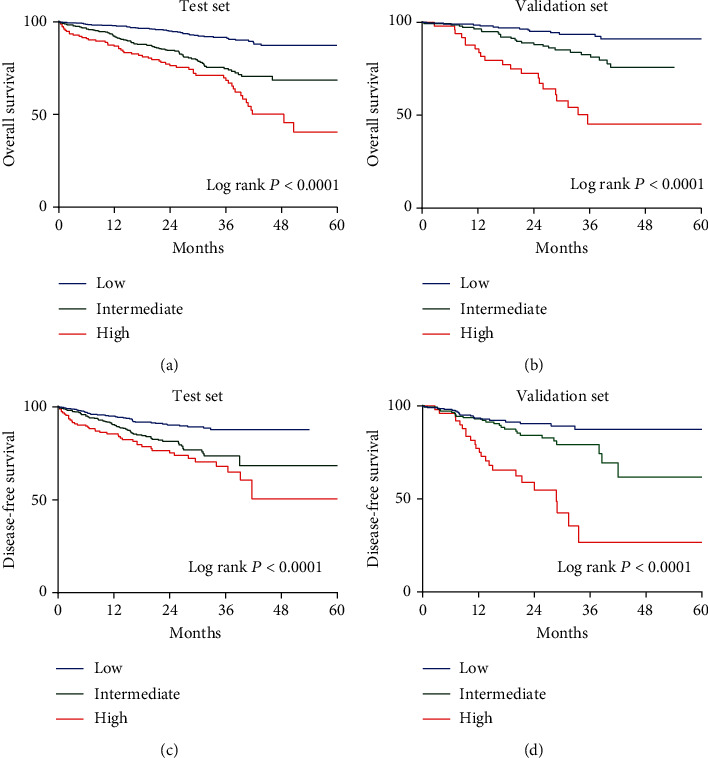
Kaplan-Meier survival plots comparing OS and DFS stratified by CRC-Integrated Oxidative Stress Score (CIOSS). OS: (a) training cohort (log rank *P* < 0.001) and (b) validation cohort (log rank *P* < 0.001); DFS: (c) training cohort (log rank *P* < 0.001) and (d) validation cohort (log rank *P* < 0.001).

As clearly shown in Table 1, we observed no significant differences in the clinical characteristics between the data sets. For the creation of CIOSS, we selected only informational oxidative stress indicators that significantly correlated with OS or DFS by performing Cox regression. As shown in Table 2, ALB, DBIL, and BUN were found to be independent risk factors for poor OS. Hence, based on the *β* coefficient, the prognostic model CRC-Integrated Oxidative Stress Score (CIOSS) was generated: 0.074 × albumin (g/L), −0.094 × bilirubin (*μ*mol/L), and −0.099 × BUN (mmol/L). Using 50% (1.60) and 85% (2.10) of CIOSS as cutoff values, three prognostically distinct groups were obtained. Kaplan-Meier survival plots comparing OS and DFS stratified by CIOSS are shown in Figures 2(a)–2(d).

### 3.3. Univariate and Multivariate Analyses of Factors Associated with OS and DFS

Univariate regression analysis was performed for all factors influencing the prognosis of patients. Then, multivariate Cox regression analysis was performed on the clinical features that were found to be significant in the univariate log-rank test. The results confirmed that TNM (hazard ratio [HR]: 3.44; confidence interval [CI]: 1.37-8.63; *P* = 0.009), T stage (HR: 2.79; CI: 1.10-7.09; *P* = 0.031), chemotherapy (HR: 0.72; CI: 0.52-0.98; *P* = 0.045), and CIOSS (Intermediate, HR: 2.93, CI: 1.99-4.32, and *P* < 0.001; High, HR: 4.33, CI: 2.80-6.68, and *P* < 0.001) were independent prognostic factors for OS (Table 3).

Furthermore, TNM (HR: 13.09; CI: 3.63-47.19; *P* < 0.001), T stage (HR: 6.8; CI: 1.60-28.87; *P* = 0.009), and CIOSS (Intermediate, HR: 1.88, CI: 1.29-2.76, and *P* = 0.001; High, HR: 3.02, CI: 1.96-4.64, and *P* < 0.001) were independent prognostic factors for DFS as well (Table 4).

### 3.4. Comparisons of the CIOSS and TNM Stage

**Figure 3:**
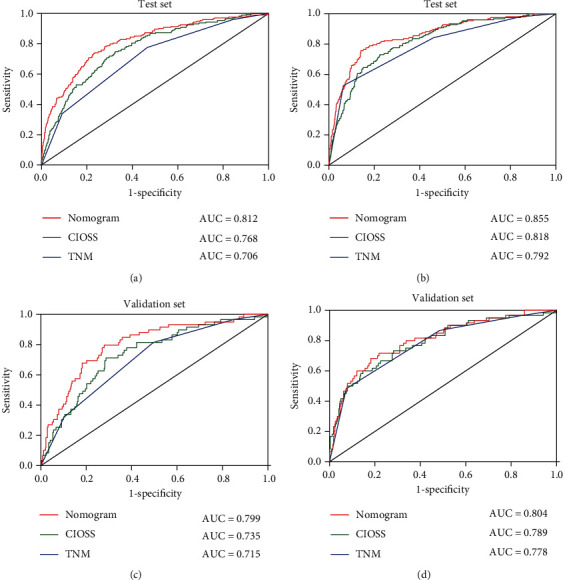
Receiver operating characteristic (ROC) curves of CIOSS, TNM stage, and the nomogram. The prognostic significance and predictive performance of the CIOSS, TNM stage, and nomogram in predicting OS and DFS in the (a, b) training sets and (c, d) validation sets, respectively.

ROC analyses were further applied to assess the predictive significance of the CIOSS in predicting survival and comparing to TNM stage. The predictive ability of CIOSS as measured by AUC in predicting OS was 0.768 in the training set (Figure 3(a)) and 0.735 in the validation set (Figure 3(c)). Similarly, the CIOSS also showed remarkable performance in the prediction of DFS among CRC patients, as reflected by the AUC of 0.818 in the training set (Figure 3(b)) and 0.789 in the validation set (Figure 3(d)). The AUCs of TNM for OS were 0.706 in the training set and 0.715 in the validation set (Figure 3). The AUCs of TNM for DFS were 0.792 in the training set and 0.778 in the validation set. Thus, we concluded that the performance of the CIOSS in predicting both OS and DFS was superior to TNM.

### 3.5. Construction and Validation of a Survival Nomogram

**Figure 4:**
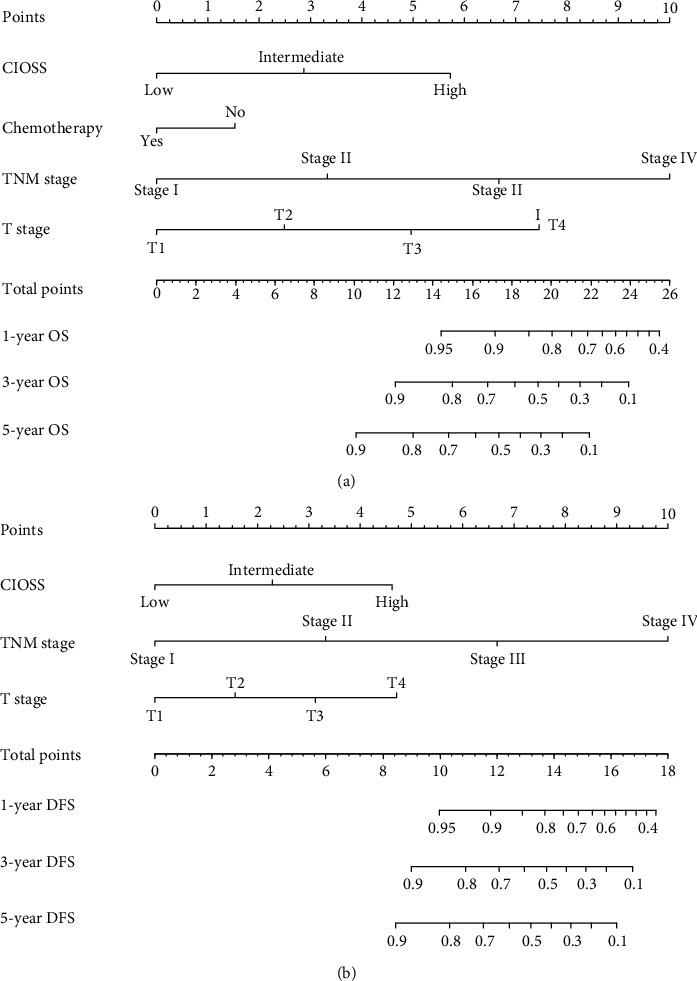
Evaluation of overall survival (OS) and disease-free survival- (DFS-) associated nomograms for resectable patients with colorectal cancer (CRC). (a) OS nomogram integrating the TNM stage, T stage, CIOSS, and chemotherapy for predicting 1-, 3-, and 5-year OS rates. (b) DFS nomogram integrating TNM stage, T stage, and CIOSS for predicting 1-, 3-, and 5-year DFS rates.

**Figure 5:**
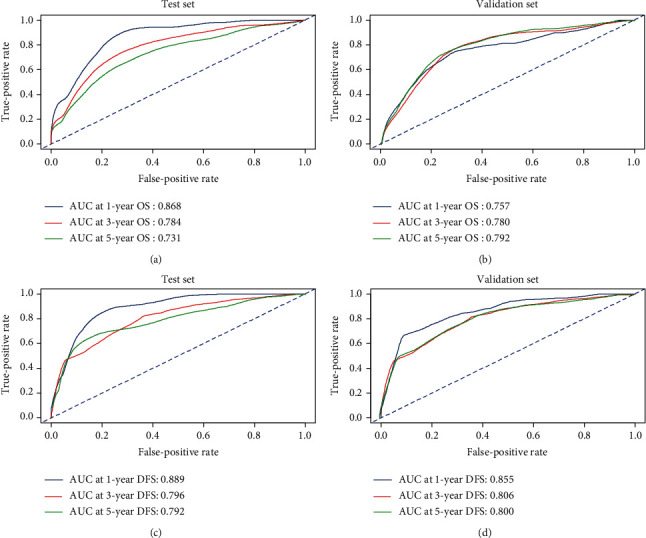
Nomograms of time-dependent receiver operating characteristic (ROC) curves associated with overall survival (OS) and disease-free survival (DFS). (a, c) Represent ROC curve of nomogram 1-, 3-, and 5-year OS rates for the training set and validation set; (b, d) represent ROC curve of nomogram 1-, 3-, and 5-year DFS rates for the training set and validation set.

Based on the multivariate Cox results for OS, four valuable factors, including TNM, T stage, chemotherapy, and CIOSS, were eventually selected to establish the predictive model (Figure 4(a)). Time-dependent- (TD-) receiver operating characteristic (ROC) analysis was used to estimate the efficiency of nomogram in predicting one-year, three-year, and five-year OS. When evaluating one-year, three-year, and five-year survival rates, the predictive power of survival nomograms measured by area under the ROC curve (AUC) in the training set was 0.868, 0.784, and 0.731, respectively (Figure 5(a)); in the validation set, it was 0.757, 0.780, and 0.792, respectively (Figure 5(b)).

For DFS, three valuable factors in the multivariate regression, including TNM, T stage, and CIOSS, were selected to establish the predictive model (Figure 4(b)). TD-ROC analysis was used to estimate the efficiency of nomogram in predicting one-year, three-year, and five-year DFS. When evaluating one-year, three-year, and five-year survival rates, the predictive power of survival nomograms measured by AUC in the training set was 0.889, 0.796, and 0.792, respectively (Figure 5(c)); in the validation set, it was 0.855, 0.806, and 0.800, respectively (Figure 5(d)).

**Figure 6:**
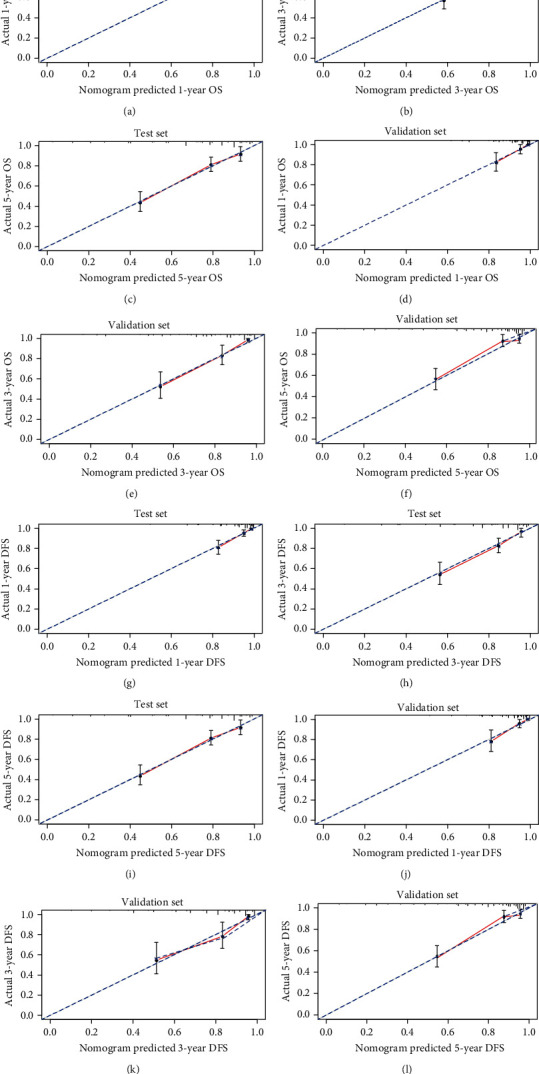
Calibration curves for 1-, 3-, and 5-year nomogram predictions. The calibration curves for predicting overall survival (OS) in colorectal cancer (CRC) patients at (a) 1, (b) 3, and (c) 5 years in the training set and at (d) 1, (e) 3, and (f) 5 years in the validation set. The calibration curves for predicting disease-free survival (DFS) in CRC patients at (g) 1, (h) 3, and (i) 5 years in the training set and at (j) 1, (k) 3, and (l) 5 years in the validation set.

The calibration curves of the two nomograms for the probability of OS and DFS in the CRC showed good agreement between prediction and observation in the test and validation sets (Figures 6(a)–6(l)). Survival nomographs based on COISS and other important characteristics have better ability to distinguish between high-risk and low-risk CRC patients in terms of OS and DFS.

## 4. Discussion

In this study, we established the oxidative stress score for the first time to predict the prognosis of CRC patients. Oxidative stress is involved in the development of a variety of cancers, including CRC; whether it also plays a role in CRC prognosis is not clear. We assessed the relationship between biochemical indicators of oxidative stress and the prognosis of CRC. In establishing a predictive model of CRC comprehensive oxidative stress score (CIOSS), we found that patients with high CIOSS had worse OS (HR = 4.33; 95% CI, 2.80-6.68; *P* < 0.001) and worse DFS (HR = 3.02; 95% CI, 1.96-4.64; *P* < 0.001) compared with CRC patients with low CIOSS. We also observed that TNM and T stages are strongly related to the prognosis of CRC and that the inclusion of these indicators together can improve the prediction of CRC prognosis significantly. The predictive nomogram had good calibration and discrimination, with an AUC of 0.731 in the test set and 0.792 in the validation set on the five-year OS. In addition, the ROC curve prediction was 0.792 in the test set and 0.800 in the validation set for the five-year DFS. This suggests that oxidative stress has an important influence on premature death in CRC patients and shows the great potential of these biomarkers have in enhancing the prediction of CRC prognosis based on tumor stage.

It is believed that the development of cancer is the result of the damage of antioxidant system, high oxidative stress will increase the risk of colorectal cancer, and autophagy and reactive oxygen species in oxidative stress can cause genetic instability and posttranslational modification of cancer-related proteins, leading to the development of CRC [13, 14].

Autophagy is a core component of the comprehensive stress response and affects the development of colorectal cancer; it has been found that BRG1 isolates and alleviates colon inflammation and tumorigenesis through autophagy-dependent oxidative stress [15]. Port et al. found that oxidative stress was associated with the aggressiveness and poor prognosis of colorectal cancer. This study found that NUAK1 is a key component of the antioxidant stress response pathway, and the absence of NUAK1 can inhibit the formation of colorectal tumors. After oxidative stress, the expression of NUAK1 in colorectal cancer is increased, leading to a decrease in the invasive height and overall survival rate of colorectal cancer [12]. Studies have found that the clearance of oxidative stress compounds is crucial to protect the body from malignant tumors. In mouse cancer cell lines and E-coli-related cancer models, it was found that after oxidative stress-related pathways were abnormally regulated, GPRC5A deletion would reduce cell proliferation, increase cell apoptosis, and inhibit the occurrence of tumors in vivo. Studies have shown that GPRC5A is a potential biomarker for colon cancer and promotes the occurrence of tumors in colitis-associated cancers by stimulating vanin-1 expression and oxidative stress [16]. Inhibiting oxidative stress in a rat model of colon cancer helps to inhibit the risk of CRC and has the potential to further evaluate therapeutic objectives [17]. In a clinical study, antioxidant enzyme activity in CRC patients was significantly lower than that in the normal population (*P* < 0.01). Assessment of oxidative stress and antioxidant administration are of great significance for the treatment and prevention of colorectal cancer [18]. Although current studies have found that oxidative stress is closely related to the occurrence and development of CRC, few studies have used the state of oxidative stress to establish CRC prediction model, and oxidative stress may be a potential indicator to improve the prediction accuracy of CRC prognosis.

In this study, we designed a new score, CIOSS, developed from a series of oxidative stress indicators significantly associated with survival in CRC patients and found that CIOSS was independent prognostic factors among patients with CRC. To build a highly accurate OS prediction model, important parameters from the univariate regression were further used for the multiple regression model. Four valuable factors, TNM, T staging, chemotherapy, and CIOSS, were selected to establish the prediction model. In this study, ROC was compared with nomogram, CIOSS, and TNM. AUC in the OS test set was 0.813, 0.768, and 0.706, respectively, and 0.855, 0.818, and 0.792 in the validation set, respectively. The AUC in the DFS test set was 0.799, 0.735, and 0.715, respectively, and 0.804, 0.789, and 0.778 in the validation set. The nomogram and CIOSS showed good discrimination and calibration and had important clinical application.

At present, many studies also use relevant indicators to predict CRC prognosis and establish corresponding prediction models. Li et al. also established a nomogram model to predict CRC prognosis based on the NLR, PLR, lymphocytes and monocyte ratio (LMR), and albumin/globulin ratio (AGR) for five-year OS and DFS [19]. They found that NLR > 2.72, PLR > 219.00, 2.83, and AGR < 1.50 were associated with a significant reduction in DFS and OS (*P* < 0.001), and the Harrell's C-indexes for OS and DFS prediction were 0.765 and 0.735. In this study, an obvious limitation of this model is that it has not been effectively verified externally, so it needs further verification. There is another multicenter retrospective study using postoperative carcinoma embryonic antigen (CEA) level, depth of tumor invasion (T factor), lymph node metastasis (N factor), and number of metastatic organs as variables, and predicting OS with postoperative CEA level, T factor, and peritoneal metastasis using factor N as a useful tool for postoperative monitoring of stage IV CRC patients, the nomograms showed c-indices of 0.629 and 0.640 in the derivation set and 0.604 and 0.637 in the validation set for DFS and OS, respectively [20]. This study is also an effective model based on common laboratory examination indicators, but its accuracy is slightly lower than that of our study. A few CRC prediction models with large sample data have also been established, and their validity has been verified both internally and externally. Boakye et al. used the Cox model and predefined variables (age, sex, stage, tumor location, and comorbidity score) to construct nomograms of relevant survival outcomes, which were found to significantly improve the prediction of CRC prognosis [21]. Li et al. established nomogram prediction for progression based on inflammatory biomarkers to predict the prognosis of CRC patients, and the results showed that the inflammatory factors included in these nomograms predicted accurate individual prognosis [22]. In addition to the combination of commonly used laboratory examination indicators into a prediction model, there have also been studies to predict the prognosis of CRC patients by combining laboratory examination and imaging examination into a prediction model. Huang et al. included a panel of radiology characteristics in their study where CT reports the status of lymph nodes and independent clinical risk factors, and through the group of radiology nomogram, model identification and calibration and applied in the validation queue nomogram still have good identification, the model showed good discrimination, with a C-index of 0.736 through internal validation, and in the validation cohort, it still gave good discrimination with C-index of 0.778 and good calibration [23]. The results of this study are of great significance, imaging examination and laboratory examination-related indicators are combined, and the prediction model is effectively validated both internally and externally. Although the accuracy of the prediction results is slightly lower than that of our study, it provides us with a good reference and suggestion. It is worth further exploring whether the integration of our research indicators into imaging examination can significantly improve the prediction effect. At present, different indexes are included in the prognosis prediction model for CRC patients, and the prediction effect of each is different. However, a reasonable and effective prediction model still needs further study.

In our study, the independent predictors of OS and DFS in multivariate analysis of CRC patients were mainly related to T stage, TNM stage, adjuvant chemotherapy, and CIOSS. Tumor stage and chemotherapy have been previously validated as independent prognostic factors for CRC patients after surgery [24, 25]. CIOSS comprised the ALB, DBIL, and BUN, which are closely related to oxidative stress and play an important role in tissue damage and disease progression. An animal model experiment found that when the oxidative stress reaction was triggered in mice after receiving external stimulation, the oxidative stress factors increased and biochemical examination showed significantly high levels of TBIL, lactate dehydrogenase (LDH), CRE, and BUN [26]. In a prospective observational study, it was found that the levels of TBIL, ALB, LDH, and CRP were significantly increased in patients with oxidative stress. After antioxidant treatment, the respective scores were significantly lower in patients than those in the control group, and the incidence of mortality and sepsis was also lower [27]. A retrospective study showed that TBIL and DBIL were significantly associated with poor prognosis after surgical resection in stages II and III CRC patients, and high DBIL had higher percentage of lymph node metastasis and lymphovascular invasion as compared with low DBIL levels (*P* < 0.05) [28]. BUN and ALB have been confirmed to be closely related to the prognosis of CRC patients and are independent prognostic factors [29, 30]. In these studies, we find that the predictive value of a single indicator is limited, while the predictive value of a combination of indicators is higher. Therefore, CIOSS and nomograms were constructed to predict CRC prognosis by combining relevant biochemical examinations that can reflect oxidative stress status. This is a new prediction model, and it has been verified to be effective.

Although the sample size of our study is large, this clinical study has some limitations. First, this retrospective study was conducted at a single center and lacked external validation. Second, although we systematically collected the laboratory examination information of patients, the levels of relevant laboratory indicators changed over time and no continuous dynamic monitoring was carried out. Finally, due to the clinical limitations, the oxidative stress indicators were not fully included. Large-scale clinical studies with more clinical and genetic characteristics conducted in multiple centers are needed to verify our results. Although there are some shortcomings, our research also has great application value. We used some common laboratory indicators to evaluate patients' oxidative stress status, which is more convenient to use and has high predictive effect and value. To our knowledge, few studies have established a CRC prognosis model from the direction of oxidative stress. This is the first time to predict the prognosis of CRC patients from the direction of oxidative stress, and our study can provide a new idea for the prediction of CRC prognosis.

## 5. Conclusions

CIOSS is a CRC-specific prognostic index based on a combination of available oxidative stress indexes. High CIOSS was found to be a powerful indicator of poor prognosis. The CIOSS also showed better predictive performance compared to TNM stage in CRC patients. The survival nomogram generated by combining the CIOSS with other beneficial clinical characteristics can potentially be a convenient and effective tool for predicting the prognosis of CRC patients.

## Figures and Tables

**Table 1:** Clinicopathological characteristics of all patients.

Characteristics	Test set (*n*=1022), %	Validation set (*n*=400), %	*P*
Age (years)			0.557
≥60	478 (46.8)	194 (48.5)	
<60	544 (53.2)	206 (51.5)	
Sex, male	590 (57.7)	235 (58.8)	0.726
Primary site			0.587
Left colon	310 (30.3)	124 (31.0)	
Right colon	197 (19.3)	83 (20.8)	
Rectum	515 (50.4)	193 (48.3)	
Family history of cancer	99 (9.7)	47 (11.8)	0.557
Histological grade			0.567
Well differentiated	153 (15.0)	60 (15.0)	
Moderately differentiated	800 (78.3)	319 (79.8)	
Poorly differentiated	69 (6.7)	21 (5.2)	
Tumor size			0.144
<2 cm	51 (5.0)	25 (6.2)	
2-5 cm	597 (58.4)	243 (60.8)	
≥5 cm	374 (36.6)	132 (33.0)	
Lymphovascular invasion			0.364
Yes	187 (18.3)	65 (16.3)	
No	835 (81.7)	335 (83.7)	
Circumferential resection margin			0.576
Yes	11 (1.1)	3 (0.8)	
No	1011 (98.9)	397 (99.2)	
T stage			0.505
T1	73 (7.1)	33 (8.3)	
T2	169 (16.5)	64 (16.0)	
T3	539 (52.7)	215 (53.7)	
T4	241 (23.7)	88 (22.0)	
N stage			0.065
N0	59 (5.8)	15 (3.8)	
N1	539 (52.7)	198 (49.5)	
N2	251 (24.6)	112 (28.0)	
N3	173 (16.9)	75 (18.7)	
TNM stage			0.563
Stage I	140 (13.7)	53 (13.2)	
Stage II	352 (34.4)	130 (32.5)	
Stage III	394 (38.6)	163 (40.8)	
Stage IV	136 (13.3)	54 (13.5)	
Adjuvant chemotherapy			0.529
Yes	492 (48.1)	200 (50.0)	
No	530 (51.9)	200 (50.0)	
Radiotherapy			0.499
Yes	55 (5.4)	18 (4.5)	
No	967 (94.6)	382 (95.5)	
Serum albumin			0.176
≥36.3 g/L	794 (77.7)	318 (79.5)	
<36.3 g/L	228 (22.3)	82 (20.5)	
Total bilirubin			0.431
≥12.2 *μ*mol/L	389 (38.1)	159 (39.8)	
<12.2 *μ*mol/L	633 (61.9)	241 (60.2)	
Direct bilirubin			0.732
≥5.2 *μ*mol/L	227 (22.2)	105 (26.3)	
<5.2 *μ*mol/L	795 (77.8)	295 (73.7)	
Urea nitrogen			0.144
≥4.6 mmol/L	616 (60.3)	233 (58.2)	
<4.6 mmol/L	406 (39.7)	167 (41.8)	
Uric acid			0.081
≥200.0 *μ*mol/L	888 (86.9)	361 (90.3)	
<200.0 *μ*mol/L	134 (13.1)	39 (9.8)	
Overall survival months	29.5 (21.8, 40.1)	29.6 (22.3, 40.7)	0.198
Disease-free survival months	20.0 (12.7, 30.2)	20.2 (13.1, 30.3)	0.732
Death, *n* (%)	174 (17.0)	59 (14.8)	0.298
Recurrence, *n* (%)	152 (14.9)	60 (15.0)	0.952

**Table 2:** Cox regression models of laboratory parameters in the test set.

	Univariate			Multivariate		
	*β*	HR	*P*	*β*	HR	*P*
ALB	-0.077	0.926	<0.001	-0.074	0.916	<0.001
TBIL	0.029	1.029	0.014	0.002	1.002	0.916
DBIL	0.110	1.116	<0.001	0.094	1.099	0.023
BUN	0.129	1.1138	0.001	0.099	1.104	0.011
UA	-0.001	0.999	0.488			

CRC-Integrated Oxidative Stress Score (CIOSS) = 0.054 × ALB -0.112 × DBIL - 0.147 × BUN.

**Table 3:** Univariate and multivariate analyses of factors associated with OS.

	Univariate analysis		Multivariate analysis	
	HR (95% CI)	*P*	HR (95% CI)	*P*
Age (years)				
≥60	1.14 (0.85-1.54)	0.378		
<60	Ref.	-		
Sex, male	1.18 (0.87-1.60)	0.296		
Primary site				
Left colon	1.56 (1.11-2.20)	0.011	1.29 (0.90-1.84)	0.169
Right colon	1.73 (1.18-2.54)	0.005	1.24 (0.83-1.86)	0.302
Rectum	Ref.	-	Ref.	-
Family history of cancer	1.03 (0.63-1.71)	0.901		
Histological grade				
Well differentiated	Ref.	-		
Moderately differentiated	1.23 (0.63-2.42)	0.547		
Poorly differentiated	1.44 (0.69-3.03)	0.336		
Tumor size				
<2 cm	0.60 (0.28-1.29)	0.190	0.82 (0.36-1.86)	0.628
2-5 cm	0.61 (0.45-0.83)	0.002	0.79 (0.58-1.09)	0.147
≥5 cm	Ref.	-	Ref.	-
Lymphovascular invasion				
Yes	1.01 (0.67-1.50)	0.983		
No	Ref.	-		
Circumferential resection margin				
Yes	0.62 (0.09-4.41)	0.630		
No	Ref.	-		
T stage				
T1	Ref.	-	Ref.	-
T2	0.83 (0.31-2.22)	0.714	0.84 (0.30-2.37)	0.748
T3	1.70 (0.74-3.91)	0.212	1.30 (0.51-3.29)	0.583
T4	5.38 (2.35-12.32)	<0.001	2.79 (1.10-7.09)	0.031
N stage				
N0	Ref.	-	Ref.	-
N1	0.56 (0.28-1.15)	0.114	0.65 (0.31-1.35)	0.245
N2	1.51 (0.74-3.05)	0.256	1.29 (0.59-2.83)	0.530
N3	2.86 (1.42-5.76)	0.003	1.82 (0.84-3.96)	0.132
TNM stage				
Stage I	Ref.	-	Ref.	-
Stage II	1.77 (0.78-4.02)	0.170	0.81 (0.32-2.08)	0.668
Stage III	4.07 (1.87-8.82)	<0.001	1.07 (0.42-2.71)	0.892
Stage IV	10.47 (4.78-22.93)	<0.001	3.44 (1.37-8.63)	0.009
Adjuvant chemotherapy				
Yes	0.66 (0.49-0.90)	0.008	0.72 (0.52-0.98)	0.045
No	Ref.	-	Ref.	-
Radiotherapy				
Yes	0.98 (0.48-1.98)	0.945		
No	Ref.	-		
CIOSS				
Low	Ref.	-	Ref.	-
Intermediate	3.08 (2.12-4.48)	<0.001	2.93 (1.99-4.32)	<0.001
High	5.09 (3.40-7.64)	<0.001	4.33 (2.80-6.68)	<0.001

**Table 4:** Univariate and multivariate analyses of factors associated with DFS.

	Univariate analysis		Multivariate analysis	
	HR (95% CI)	*P*	HR (95% CI)	*P*
Age (years)				
≥60	1.13 (0.82-1.55)	0.457		
<60	Ref.	-		
Sex, male	1.02 (0.74-1.41)	0.886		
Primary site				
Left colon	1.40 (0.98-2.02)	0.068	1.15 (0.79-1.67)	0.481
Right colon	1.52 (1.01-2.29)	0.048	1.26 (0.82-1.93)	0.303
Rectum	Ref.	-		
Family history of cancer	1.26 (0.70-2.26)	0.450		
Histological grade				
Well differentiated	Ref.	-		
Moderately differentiated	1.31 (0.81-2.12)	0.279		
Poorly differentiated	1.50 (0.73-3.09)	0.272		
Tumor size				
<2 cm	0.71 (0.31-1.64)	0.421		
2-5 cm	0.88 (0.63-1.23)	0.453		
≥5 cm	Ref.	-		
Lymphovascular invasion				
Yes	0.96 (0.62-1.48)	0.845		
No	Ref.	-		
Circumferential resection margin				
Yes	1.49 (0.37-6.03)	0.573		
No	Ref.	-		
T stage				
T1	Ref.	-	Ref.	-
T2	2.21 (0.49-9.96)	0.303	2.93 (0.63-13.55)	0.170
T3	4.39 (1.07-17.92)	0.040	3.71 (0.88-15.73)	0.075
T4	13.23 (3.25-53.88)	<0.001	6.80 (1.60-28.87)	0.009
N stage				
N0	Ref.	-	Ref.	-
N1	0.83 (0.35-1.93)	0.659	1.02 (0.43-2.43)	0.958
N2	1.87 (0.80-4.38)	0.152	1.30 (0.53-3.19)	0.570
N3	4.00 (1.71-9.23)	0.001	1.88 (0.78-4.51)	0.160
TNM stage				
Stage I	Ref.	-	Ref.	-
Stage II	2.63 (0.78-8.81)	0.118	1.38 (0.37-5.16)	0.634
Stage III	5.64 (1.76-18.09)	0.004	2.24 (0.61-8.18)	0.223
Stage IV	33.45 (10.56-105.93)	<0.001	13.09 (3.63-47.19)	<0.001
Adjuvant chemotherapy				
Yes	0.82 (0.68-1.17)	0.328		
No	Ref.	-		
Radiotherapy				
Yes	0.51 (0.29-0.91)	0.022	0.92 (0.51-1.67)	0.794
No	Ref.	-	Ref.	-
CIOSS				
Low	Ref.	-	Ref.	-
Intermediate	2.17 (1.49-3.16)	<0.001	1.88 (1.29-2.76)	0.001
High	3.09 (2.02-4.71)	<0.001	3.02 (1.96-4.64)	<0.001

## Data Availability

The data used to support the findings of this study are available from the corresponding author upon request.

## Supplementary Materials

Supplementary Materials The supplementary pictures have been uploaded to the attachment. Fig S1: the oxidative stress indicators were statistically associated with OS and DFS in CRC patients. The curves of ALB, TBIL, DBIL, BUN, and UA for OS in CRC patients (A-E); the curves of ALB, TBIL, DBIL, BUN, and UA for DFS in CRC patients (F-J).
