# Supplementary figures and images for: An Oxidative Stress Index-Based Score for Prognostic Prediction in Colorectal Cancer Patients Undergoing Surgery
Source: Oxid Med Cell Longev. 2021 Jan 9;2021:6693707. doi: 10.1155/2021/6693707 (PMC7811428; doi:10.1155/2021/6693707)

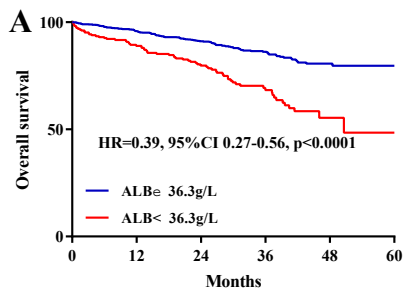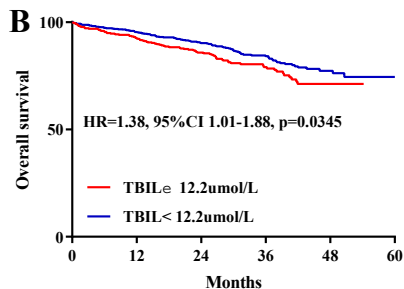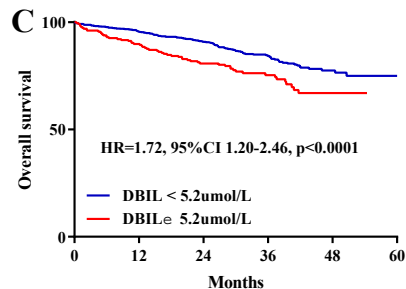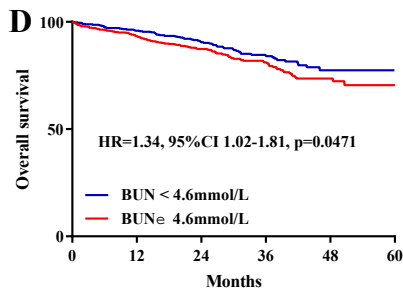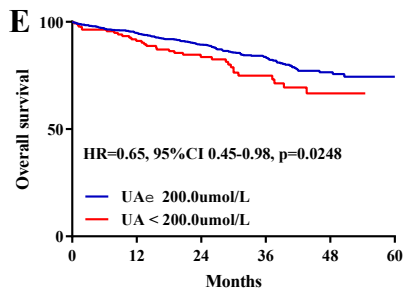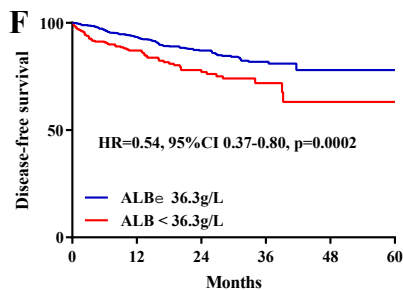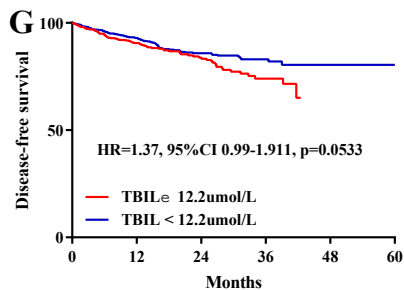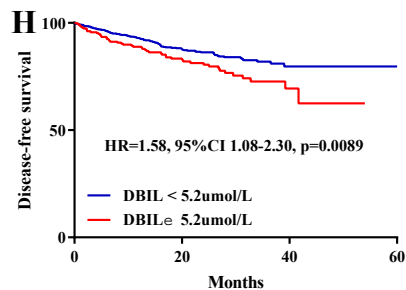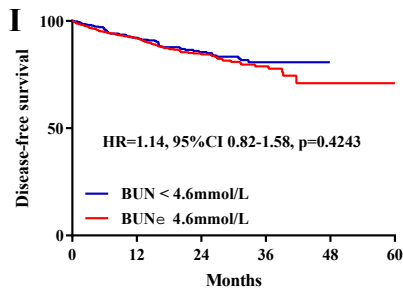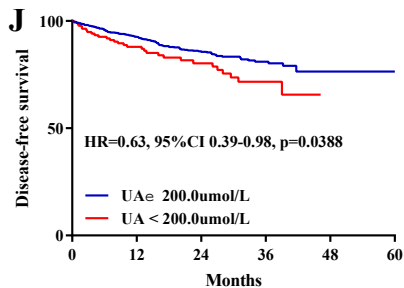

Supplement: Supplementary Materials — The supplementary pictures have been uploaded to the attachment. Fig S1: the oxidative stress indicators were statistically associated with OS and DFS in CRC patients. The curves of ALB, TBIL, DBIL, BUN, and UA for OS in CRC patients (A-E); the curves of ALB, TBIL, DBIL, BUN, and UA for DFS in CRC patients (F-J). [file 6693707.f1.pdf]
